# Study on Health Monitoring and Fatigue Life Prediction of Aircraft Structures

**DOI:** 10.3390/ma15238606

**Published:** 2022-12-02

**Authors:** Yanjun Zhang, Bintuan Wang, Yu Ning, Haifeng Xue, Xiaoxin Lei

**Affiliations:** AVIC the First Aircraft Institute, Xi’an 710089, China

**Keywords:** structural health, individual aircraft tracking, life prediction, fatigue, load spectrum

## Abstract

In this paper, some progress and achievements in aircraft integrity requirements, structural health monitoring, load spectrum measurement and life assessment research were presented. Several concepts of structural health monitoring were analyzed and compared, and the basic flow chart for health monitoring and life prediction of an aircraft structure was given. The selection of control points, construction of load/strain equations and stress calculation of control points were also described. Reliable IAT (Individual Aircraft Tracking) and life monitoring methods and software for IAT were developed for a certain type of aircraft, and fatigue life prediction of an aging aircraft was conducted based on actual measurement of load spectrum. The main features such as damage calculation, life evaluation and result output were discussed, and the future research focuses relating to intelligent structural health monitoring were finally explored.

## 1. Introduction

The technology of Prognostics and Health Management (PHM), which can realize the transformation of aircraft from traditional health monitoring to new health management, and ensure the system structure safety, performance integrity, economy and safety in the life cycle, has gradually become the key technology for compressing maintenance cost, supporting equipment to achieve high efficiency and self-health management [1].

As one of the important components of PHM, aircraft Structural Health Monitoring (SHM) can play an important role in the design, flight and maintenance of aircraft. Information of structural response, operation and service environment can be obtained through the built-in sensor network in the aircraft structure. The diagnosis results based on the sensor data can be used for an initial judgment on the structural health status and so, for decision-making about auxiliary maintenance and maintenance [2].

**Figure 1:**
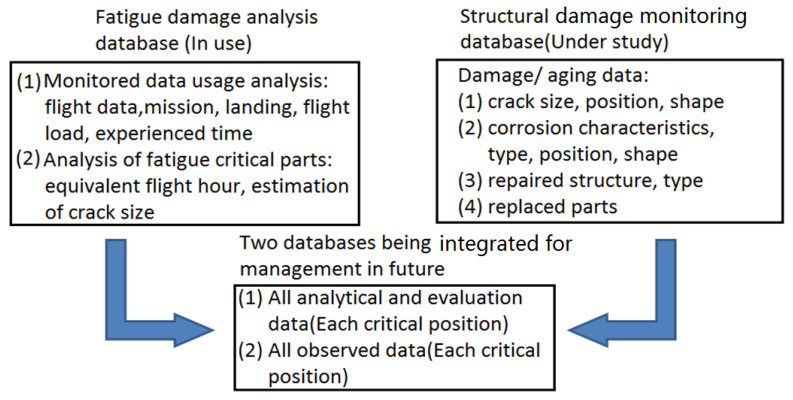
US air force aircraft management schematic [3].

The aircraft management path map of the US Air Force is shown in Figure 1 [3]. The future development direction of air force aircraft management is to combine structural damage monitoring data with structural fatigue damage analysis data, and to establish a data-based aircraft structural life management system by means of “virtual–real integration”.

Based on the analysis of the requirements for structural health monitoring by military aircraft development standards, this paper summarizes the typical engineering cases of structural health monitoring in other countries, and describes the research on the life tracking of individual aircraft with center-of-gravity overload along with some advanced structural health monitoring system. The processes of health monitoring and life prediction of aircraft structure are presented. The selection of control points, construction of load/strain equation, stress calculation of control points, damage calculation, life evaluation and output of results are described. Finally, the development direction from the present design technology to the future intelligent application is examined.

## 2. Basic Theory of Structural Health Monitoring

### 2.1. Different Expressions of Structural Health Monitoring

In general, when it comes to structural health monitoring, the following phrases are common in the literature:

1. Prognostics and Health Management, or PHM [1];
2. Integrated Vehicle Health Management, or IVHM [4];
3. Structural Health Monitoring/Management, or SHM [2];
4. Structure Prognostics and Health Management, or SPHM [5];
5. Usage Monitoring Function, or UMF [6];
6. Individual Aircraft Tracking, or IAT [7,8];
7. Prognostic and Probabilistic Individual Aircraft Tracking, or P2IAT [9].

Generally, PHM and IVHM are defined from a relatively general and systematic point of view in order to describe the aircraft’s capability status relative to its overall capability state (including structure, system, etc.). In particular, PHM is more usually used in failure prediction, while IVHM has a larger connotation and scope.

SHM and SPHM were evolved from the PHM concept, focusing on fault prediction and health management-related descriptions of aircraft structures. Both of these concepts are common in the literature, but it is generally accepted that SHM is more versatile and is commonly used for abbreviation of Structural Health Monitoring rather than Structural Health Management.

IAT is the basic requirement for the implementation of the Force Management in the five tasks of the Aircraft Structural Integrity Outline, aiming at determining and adjusting the inspection and maintenance intervals based on the actual data measured on an individual aircraft.

Since 2013, the US Air Force has launched the Aircraft Digital Twin (ADT) program, focusing on the development of a new IAT framework, known as Prognostic and Probabilistic Individual Aircraft Tracking (P2IAT), to replace the current benchmark deterministic IAT framework. In particular, P2IAT is more probabilistic (or uncertain), diagnostic, and predictive than current IAT methods.

The Usage Monitoring Function (UMF) is used in small amounts in the structural health monitoring for Airbus. For example, the UMF equipment was developed for A400M aircraft.

PHM idea was first formed from aircraft structural life monitoring and management, and SHM was developed from individual aircraft fatigue life monitoring technology. The real-time tracking of high-value aircraft platform, fault diagnosis, future prediction, and safe operation of aircraft are the essential purposes of both structural health monitoring and structural prognostics and health managements. It can reasonably guide the aircraft operation and decision for structure maintenance, and it will also improve the aircraft inspection maintenance from being on a scheduled basis to a condition basis.

Therefore, the differences between the above-mentioned expressions of SHM are more reflected in the usage habits and application scenarios. It is recommended that the term structural health monitoring (SHM) be used when talking about the aircraft structural health monitoring technology, and individual aircraft tracking (IAT) when more emphasis is being placed on aircraft usage tracking and life consumption assessment.

### 2.2. Requirements of Design Specification

The development of the latest model has put structural health monitoring into the development requirements [3,7,10].

The MIL-STD-1530D [7], released in 2016, requires the establishment of IAT system, consisting of hardware, flight data download equipment, data processing, data conversion and software that can be used to adjust the inspection interval of individual aircraft. At the same time, it is required that all military aircraft should be able to record sufficient service parameters to determine the damage growth rate of structures and factors affecting service life. The system should be at least capable and reliable enough to record a minimum of 90% of all data in the aircraft’s lifetime.

The American Aerospace Industry Commission has organized a number of organizations and agencies, including Airbus, Boeing, the US Air Force and Navy, NASA, the European Aviation Safety Agency and Stanford University, to jointly develop the ARP6245-Military Aircraft Structural Health Monitoring Standard [11] to standardize its aeronautical application research and development.

The GJB 67.6A-2008 standard on “Repetitive Load, Durability and Damage Tolerance” [12] proposes that “the user should complete the load/environment spectrum measurement, individual aircraft tracking data, individual aircraft maintenance time, structure maintenance record and so on during the use of the force to ensure the damage tolerance and durability of each aircraft”.

GJB775A-2012 “Military Aircraft Structural Integrity Outline” [7] calls for “the establishment of procedures and methods for the use of troops to ensure the structural integrity of each aircraft throughout its lifetime” and explicitly calls for “the establishment of an individual aircraft tracking outline”. Structural Integrity is defined as “the state in which the structure can be used normally and when the function is not weakened at the required level of structural safety, structural capability, durability and affordability, including the strength, stiffness, durability, damage tolerance and function of the aircraft that affect the safe use and cost of the aircraft”.

The current domestic and international aircraft design standards do not give a specific definition of aircraft structural health. By reference to related standards, the structural health of an aircraft can be defined as the ability of the aircraft structure to maintain integrity, i.e., the normal use of the aircraft structure and the state in which the function is not impaired.

The IAT outline is based on the actual measurement data of the individual aircraft. It is mainly used to adjust the time of structural inspection, change, overhaul and replacement; to determine the damage growth under the corresponding environmental conditions during the whole operation; and to quantify the changes in the use of the mission. It can also be used to determine the equivalent flight hours and to adjust the maintenance plan for all key parts of each aircraft, as well as to predict when the life limit of the aircraft structure will be reached.

In summary, the purpose of IAT consists of the following two elements. First, the implementation of the training program of the fleet is evaluated by using monitoring and tracking to assess whether the conditions (task composition) of the design life have changed. Second, according to the above changes, the life analysis and evaluation of the fleet aircraft are carried out to optimize the assignment of the training task of the fleet aircraft and revise the structural integrity control plan [7].

### 2.3. Introduction to Typical Engineering Case of SHM

The monitoring of aircraft structural health has been studied for many years in the United States, Europe and other countries. Numerous ground tests and flight verification tests have been carried out from the perspective of the monitoring mechanism, test and ultimately verification of its airworthiness. Several complete template databases have been established for different structural parts of aircraft by means of flight measurement, finite element simulation and whole-aircraft test calibration.

From the B-1B which was first designed to record structure load histories by integrated devices to the IAT system on the F/A-18, F-15, F-16, F-111, F-22 and other aircraft, the basic principle of the system is to obtain the load environment under the actual flight conditions of the critical parts through the airborne equipment, and to carry out the individual aircraft life prediction [13,14].

Taking the F-35 and A400M aircraft as examples, the typical engineering cases of the application of structural health monitoring technology are presented below.

#### 2.3.1. Structural Health Monitoring of the F-35 Aircraft

**Figure 2:**
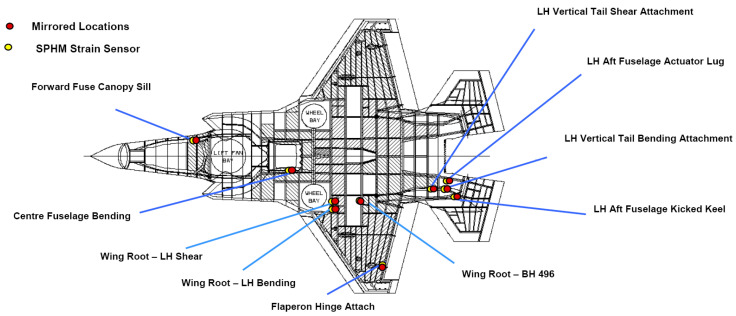
Strain gage location of F-35 [15].

The F-35 developed a complete system of Structure Prognostic and Health Management (SPHM) in the process of aircraft development, which is used for operation and life management [15]. In this system, load equation calculation and strain sensor measurement are used to obtain external load, in which the load calculation method is the principal one and strain measurement is the auxiliary method. Strain measurement is mainly used for the establishment, calibration and optimization of the load equation. Strain sensors are installed in 10 locations on one side of the aircraft (with the center line of the fuselage as the symmetrical line), and the load on the other side is calculated by the load equation. The specific distribution of strain sensors on the aircraft is shown in Figure 2.

Strain sensors were only installed on some aircraft for F-35, as follows [15]:

- Installed on all aircraft at the development stage;
- Installed on 10% of aircraft at the stage of mass production.

Strain sensors have been used until natural failure has occurred without maintenance. The local stress of each control point is calculated by the established external load function equation.

The same damage model was adopted in IAT as in the design stage. For example, the damage tolerance model is used for F-35A (CTOL, Conventional Take-Off and Landing), while the strain life method was used to calculate damage for F-35B (STOVL, Short Take-Off and Vertical Landing) and F-35C (CV, Carrier Variant).

**Figure 3:**
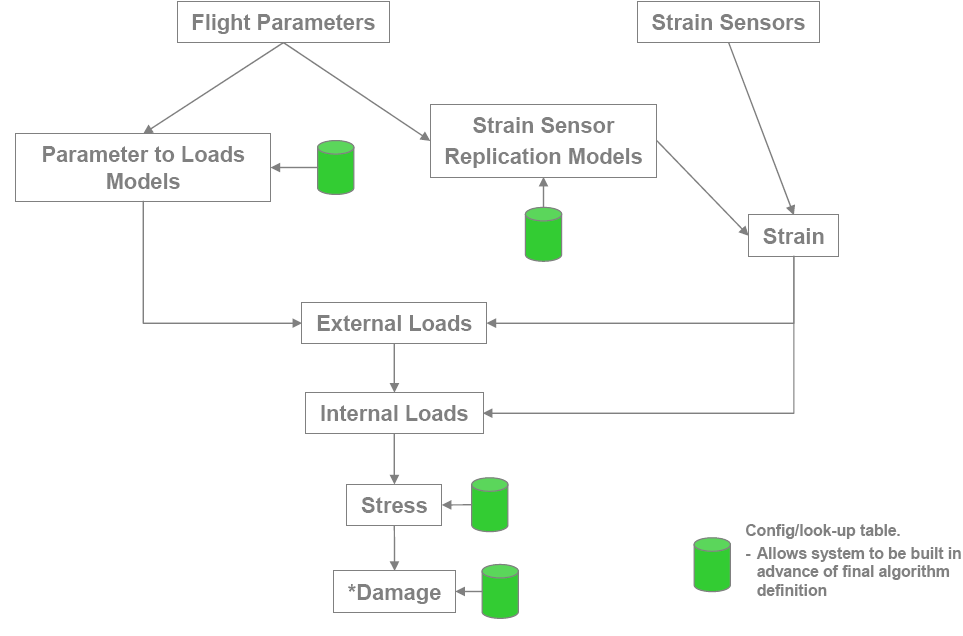
Flowchart of load monitoring for F-35.

Figure 3 shows the flow chart of F-35 aircraft load monitoring [15].

Using SPHM technology, 90% of the delivered F-35 aircraft is expected to reach 30 calendar years or 8000 flight hours.

#### 2.3.2. Structural Health Monitoring of the A400M Aircraft

The A400M is a multi-purpose military transport aircraft developed by Airbus. To cover the various types of tasks that are expected to be used during the service period, the Usage Monitoring Function (UMF) was used to carry out structural health monitoring and evaluate the fatigue damage in service for each aircraft.

The UMF of A400M aircraft includes three complementary functions [7], which are:

- Non-direct measurement function. Based on the parameters recorded by the aircraft system, the load and stress history of typical structural parts are reconstructed, and fatigue and crack propagation damage are evaluated.
- Statistical usage function. This provides the fatigue parameters derived from the actual use of each aircraft.
- Direct measurement function. This was only installed on some aircraft, including strain sensors that monitor the use of loads and cross-check the estimated load and stress history.

The UMF method adopted by A400M reconstructs the local load history of the Pilot Point (control point). By constructing the transformation equation between the recorded results and the load or stress at any time, the stress spectrum of each control point is obtained and the fatigue damage is calculated. The establishment of the conversion equation is complicated because it requires the establishment of a load model whose accuracy has to meet the requirements and can cover any flight profile.

**Figure 4:**
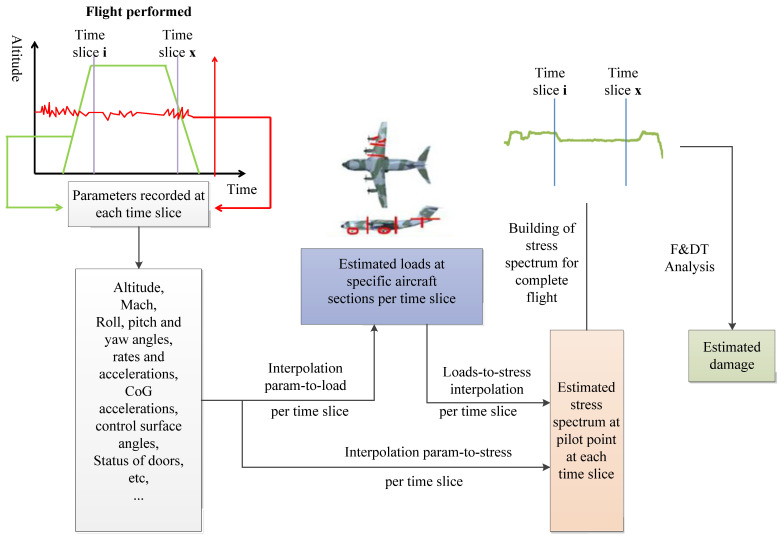
The usage monitoring function process for A400M.

Figure 4 shows the UMF flow chart of the A400M aircraft [7].

The UMF of A400M provides fatigue and crack propagation damage indices at specified control points. This damage index is defined as the ratio of actual flight damage to the average damage per flight (which can be the damage of the flight profile used for design). Based on this, the cumulative damage status of each aircraft since it has been put into service can be calculated directly.

## 3. Research and Application of Structural Health Monitoring Method

### 3.1. Related Research Progress

It is difficult to carry out IAT for aircraft in service by directly installed sensors, which can only conveniently be used when developing a new aircraft. However, based on the status of aircraft operation, the IAT research can be carried out by using the life monitoring method based on the actual flight record process of individual aircraft, and some useful progress has been made in this area. For several new aircraft, an individual aircraft life monitoring system has been established and put into practice [16,17,18,19,20,21,22].

He et al. [16] studied the scatter system of fatigue life of aircraft joints, and proposed an equivalent damage principle whereby flight hours can be converted into equivalent flight hours. The obtained safety life of aircraft structure can be used as the reference life value of individual aircraft life management. Huang et al. [17] discussed the life extension of a certain type of advanced fighter, expounded in detail the actual application of IAT technology, calculated the damage based on the center of gravity overload, worked out the corresponding IAT measures, and realized the service life management of each aircraft. Zhang et al. [18] gave the criterion formula and the corresponding analogical calculation model of each stage of aircraft fatigue life for IAT, and connected the damage of IAT with the average damage of the aircraft fleet. Finally the life loss coefficient was derived and then used to determine the service life and the residual life of individual aircraft. Yao et al. [19] studied the advantages and disadvantages of the life monitoring mode, and concluded that developing an aircraft structure health monitoring system based on intelligent materials is the future direction for life monitoring of individual aircraft. Song et al. [20] studied development of technology in aircraft structure life monitoring and management, and made a prospect of aircraft structure health monitoring systems and technology applications.

The concept of equivalent damage was introduced into the IAT and life monitoring, and the individual aircraft life monitoring technology based on equivalent damage was also developed [23]. The aircraft structure was treated as a whole, the potential growth of fatigue damage or fatigue life consumption was measured by equivalent damage, and the uniform equivalent damage growth was used to measure the life consumption of the aircraft structure.

Cusati et al. [24] studied the design guidelines for structural health monitoring systems—based on multidisciplinary analysis—that determine the break-even point through aircraft weight gain and direct operating costs. The research can maximize the benefits of this innovative technology by considering structural health status monitoring from the conceptual design stage.

The energy acquisition optimization of a reinforced composite shell in flight was studied by Daraji et al. [25]. An optimal positioning method for piezoelectric sensors based on the average percentage maximization was proposed, and the sensor efficiency was taken as the objective function. The energy acquisition of a composite wing under the structural frequency in flight was optimized. This method can reduce the computational workload and improve the efficiency of energy acquisition for complex and large structures.

Based on the traditional data and feature level fusion method, Wei et al. [26] proposed a new decision level fusion method for engine structure health monitoring. The remaining service life of an aircraft engine was evaluated by integrating multiple sensors together. The fusion method of a decision-making layer was proposed by using convex optimization to solve the problem and determining the optimal weight.

Ranasinghe et al. [27] studied the challenges of the research and application of IHM systems in aerospace, and gave a key role in future applications to network physics and autonomous systems.

Lopresto et al. [28] studied the detection of shock dynamic response and damage behavior, introduced a new method combining depth learning and wave propagation to perceive the shock response spectrum of piezoelectric plates at different frequencies, and looked forward to the implementation strategy of guided wave health monitoring.

Romano et al. [29] proposed a health monitoring system for damage identification, location and analysis of piezoelectric ceramic and optical fiber sensors in a composite wing panel, which provided a feasible direction for better weight decrease and better structure performance.

### 3.2. IAT and Life Control

#### 3.2.1. IAT Process Description

**Figure 5:**
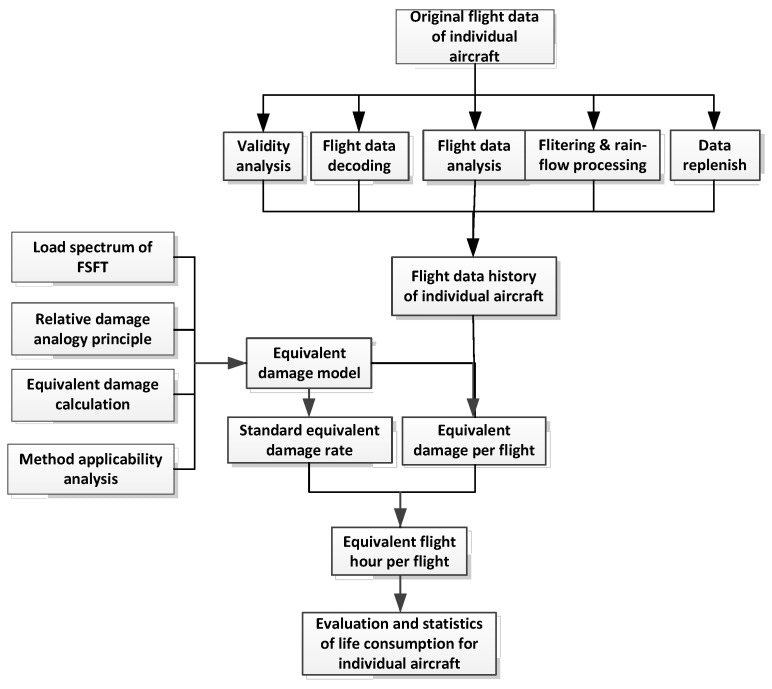
Process of individual aircraft tracking and life monitoring.

In the IAT practice for a certain type of aircraft, based on the historical flight parameter data of the individual aircraft in the field and the full-scale fatigue test spectrum of the aircraft, the relative damage analogy method was adopted to determine the equivalent damage model and damage index, which is adopted in the automatic calculation technology of flight damage parameters. Key technologies such as service life consumption assessment provide mature and reliable IAT and life monitoring methods, and a managing software for IAT. The flow chart of IAT and life monitoring is shown in Figure 5.

Considering the structural characteristics of key parts of typical aircraft, the characteristics of materials, and the load/environment spectrum experienced based on the reference spectrum, various damage calculation formulas were compared. The equation for equivalent damage calculation and the damage index based on the overload of the center of gravity were then obtained through numerical analysis. For this purpose, the reference spectrum was taken from the full-scale fatigue test load spectrum compiled from flight measurement data. On this basis, the analysis and determination of the sampling rate and filtering threshold in the IAT were presented to provide technical support for the implementation of IAT and life monitoring.

Life consumption assessment of in-service aircraft is a direct means to give the relative relationship between the use of an individual aircraft in the fleet and the basis for IAT management.

#### 3.2.2. Damage Accumulation Theory and Equivalent Damage Models

Although there are many kinds of fatigue damage calculation methods in engineering, there is still no recognized accurate and effective method for the calculation of aircraft damage, especially when there is no specific dangerous position and corresponding stress data. Miner’s law of linear damage accumulation theory has been popular in the field of industry due to its simple mathematical expression. The biggest advantage of Miner’s law is its methodology and philosophy for all of the structure, but its forecasts are dispersed and lack precision, which limits its application.

Based on relative Miner’s law, the calculation formula of the analog method used to predict aircraft life (damage) is shown as follows [30]:

(1) $$\;\lambda^{\prime }=\frac{\sum_{i=1}^{k}n_{i}{\left(\Delta g_{i}\right)}^{m}}{\sum_{k=1}^{j}n_{k}^{\prime }{\left(\Delta g_{k}^{\prime }\right)}^{m}}\lambda$$

In the formula above, *λ* is the life of an aircraft with known life; *λ*’ is the life of an aircraft with unknown life; $n_{i}$ is the repeated cyclic numbers of the load at level ith in the spectrum for the aircraft with known life; $n_{k}^{\prime }$ is the repeated cyclic numbers of the load at level kth in the spectrum for the aircraft with unknown life; $\Delta g_{i}$ is the increment of the load at level ith in the spectrum with known damage; $\Delta g_{k}^{\prime }$ is the increment of the load at level kth in the spectrum with unknown damage; and m is a constant, commonly known as the damage index.

The load spectrum of full-scale fatigue test for a certain type of aircraft was composed of 40 representative flights, which is shown in Table 1. The load spectrum was developed based on the representative flights with a random sequence of 335 flights in total and the spectrum was then used as the reference spectrum.

Goodman’s formula, which is based on the constant life curve, assumes that the fatigue strength decreases linearly with the increase in the mean stress $\sigma_{m}$. The formula is as follows:

(2) $$\sigma_{a}{}_{,i}=\sigma_{-1}\left(1-\frac{\sigma_{m}{}_{,i}}{\sigma_{b}}\right)$$

In this formula, $\sigma_{-1}$ is the stress amplitude or maximum stress corresponding to the stress ratio R = −1; $\sigma_{m,i}$ is the average stress corresponding to any load cycle; and $\sigma_{a,i}$ is the stress amplitude corresponding to any load cycle.

According to the usage, assuming that stress is linear with overload, the variation of each cycle in the spectrum is expressed as

(3) $$\Delta G_{i}={\left(G_{\max}\right)}_{i}-{\left(G_{\min}\right)}_{i}$$

The damage of each cycle $\left(\Delta G_{i},R_{i}\right)$ is converted into a pulsation cycle, and then the equivalent damage formula is established as follows [23].

(4) $$D=\sum_{i=1}^{n}{\left[{\left(G_{\max}\right)}_{0i}\right]}^{m}$$

In the formula, ${\left(G_{\max}\right)}_{0i}$ is the maximum overload of the pulsating cycle, m is the damage index, and n is the number of load cycles in the load spectrum.

Based on the damage calculation formula mentioned above, the damage of individual aircraft can be calculated and compared with the damage of the reference spectrum.

#### 3.2.3. IAT Output

**Figure 6:**
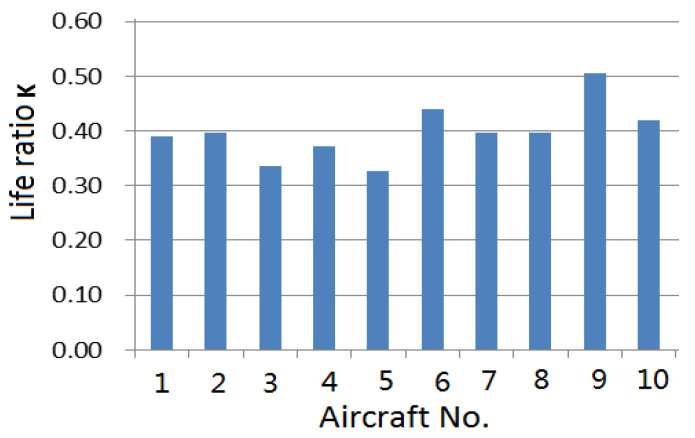
Fatigue life consumption for different aircraft in fleet.

After calculating the reference spectrum to determine the reference equivalent damage per flight hour (standard equivalent damage rate), according to the flight parameter data processing results of each aircraft, the equivalent damage corresponding to the actual flight hours was converted through the damage analogy. The individual aircraft life consumption assessment presented information about the consumption assessment of the flight life for the fleet, the annual life consumption assessment, the equivalent damage statistics of typical flight profiles, and the statistical assessment of the life ratio. Through the life ratio, the speed of individual life consumption per hour was given. The fatigue life consumption ratio is shown in Figure 6.

It can be seen from Figure 6 that the life consumption rate of different aircraft in the same fleet is different, and they are all lower than the standard equivalent damage rate.

**Figure 7:**
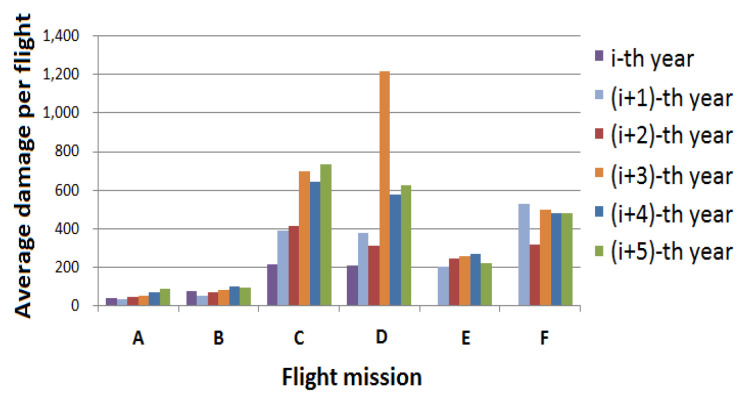
Variation of annual damage for flight subjects.

Figure 7 shows the average flight damage of five different profiles A, B, C, D, and E for 6 consecutive years. It can be seen that the equivalent damage of different profiles varies greatly with different years.

The IAT life consumption assessment can identify the degree of individual aircraft life consumption in the fleet, the degree of annual life consumption, and the life consumption of typical profiles, so as to provide a comprehensive and multi-level life assessment. For the problems existing in the life consumption of the individual aircraft, corresponding solutions are given, and specific suggestions for subsequent use are proposed.

### 3.3. Prediction of Fatigue Life of Aging Aircraft Based on Actual Measurement of Load Spectrum

**Figure 8:**
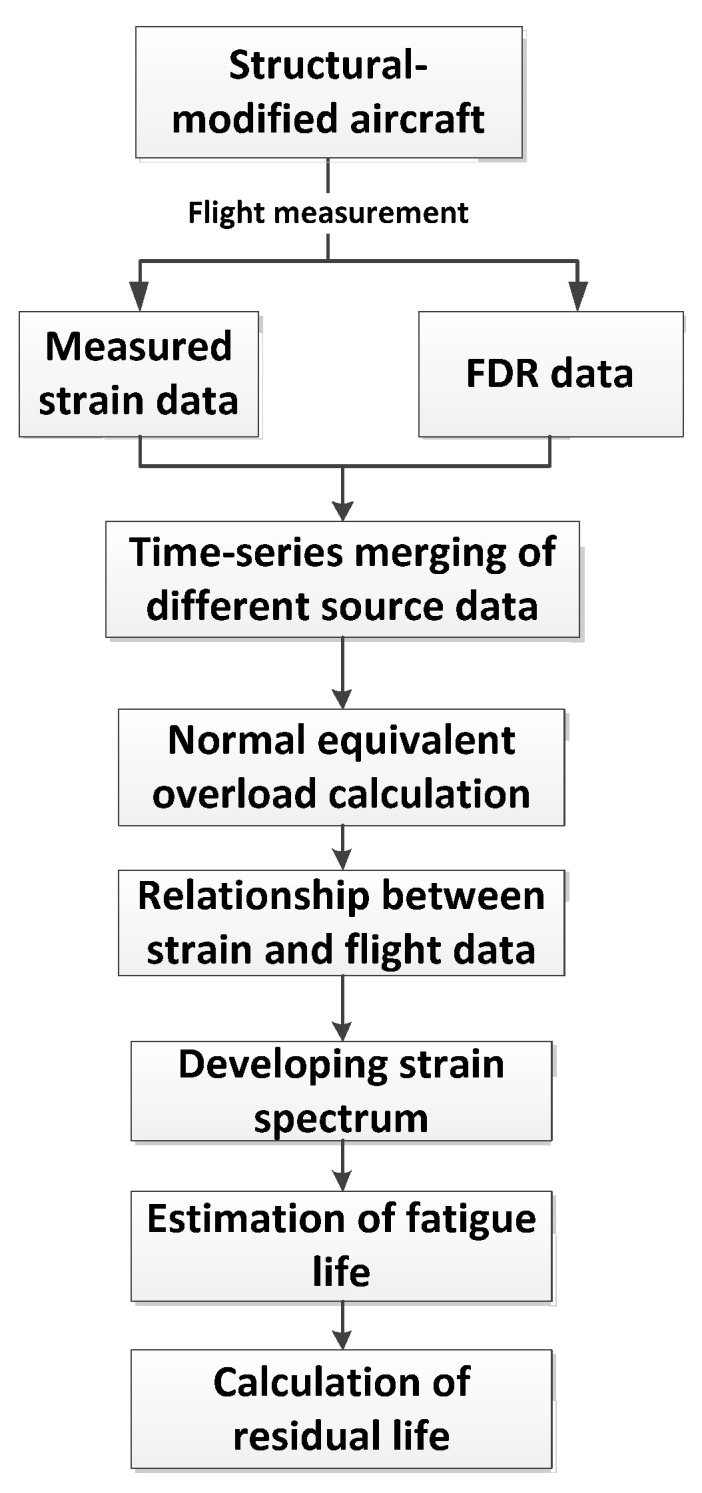
Flowchart of strain spectrum development from flight tests and fatigue life estimation for aging aircraft [31].

Aiming at the problem of the longevity of the modified structure of an aging aircraft, it was proposed to install strain gauges on the modified part of the fuselage, obtain the measured strain data of the part through actual flight measurement, and determine the technical plan of the local measured load spectrum together with the flight parameters. The road map of local load spectrum measurement and fatigue life assessment is shown in Figure 8.

**Figure 9:**
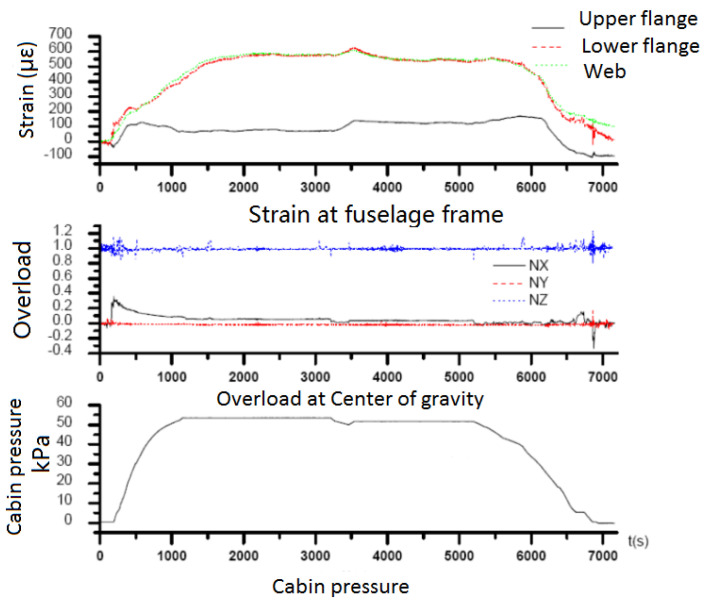
History of local strain and some flight parameters.

By combining the measured strain data of the fuselage with the flight parameter data in the actual flight measurement in terms of time and frequency, the actual fatigue load data of the modified parts of the fuselage was developed. The flight parameter data and the measured strain data were combined with the data time history. Some flight parameters and local strain history curves are shown in Figure 9; the changes of the parameters were in good conformity after testing.

For the measured strain data, the correlation analysis with the flight parameter data was carried out, and the correlation between the strain data of the modified parts of the aircraft and the flight parameter data was calculated. Seven main flight parameters—including overloads for three directions, triangular velocity, and pressure difference between inside and outside the cabin—were screened, and the relationship between the average strain data of the modified part of the aircraft and the flight parameter data of the aircraft was obtained.

On this basis, a fatigue test under the modified structure spectrum load was carried out, the results of which are shown in Table 2.

The fatigue life was assumed to be a two-parameter Weibull distribution, for which the distribution function is

(5) $$F(N)=1-\exp{\left[-{\left(\frac{N}{\beta }\right)}^{\alpha }\right]}^{}$$

Where β is characteristic life; and α is the shape parameter of material. According to the literature [32], the shape parameter is 2.0–2.5 for high strength steel, 2.5–3.0 for titanium alloy, 3.0–3.5 for low strength steel and 3.5–4.5 for aluminum alloy. According to different types of materials or structures adopted for the aircraft, the shape parameters can be obtained statistically from test data.

The characteristic life β can be calculated due to the experiment data according to literature [33]:

(6) $$\beta ={\left[\frac{1}{n}\sum_{i=1}^{n}N_{i}^{\alpha }\right]}^{\frac{1}{\alpha }}$$

The structure life of 95% confidence and different reliability obtained by experiment is shown below.

(7) $$N_{95/\gamma }=\frac{\beta }{S_{T}S_{C}S_{R}}$$

**Figure 10:**
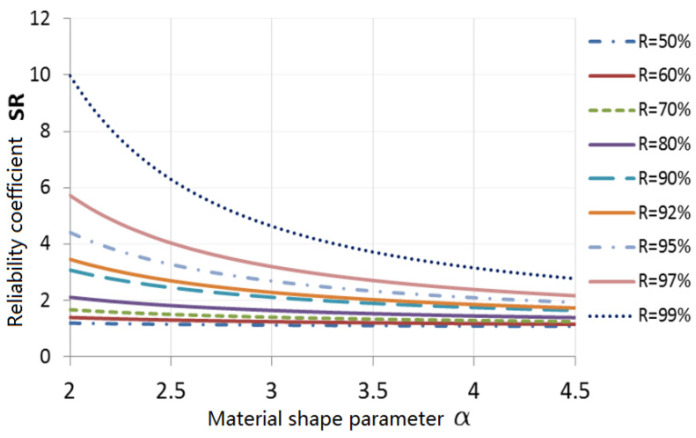
Reliability factors of different reliability levels and material shape parameter.

The specimens were manufactured from aluminum alloy and the result shows that there are seven valid samples. We were then able to determine that the coefficient of specimen was $S_{T}=1.1$ and the confidence coefficient was $S_{C}=0.97$. The reliability coefficients for different reliability and different material shape parameters are shown in Figure 10.

**Figure 11:**
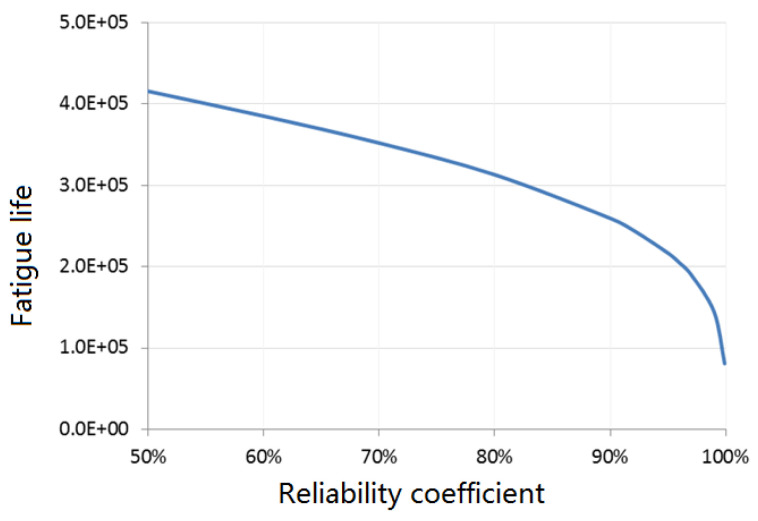
Fatigue lives under different reliability levels.

The test fatigue life corresponding to different reliability levels with 95% confidence is shown in Figure 11.

In order to ensure the safety of the aircraft, the structural life of aircraft with 99.9% reliability can be conservatively measured as 80,966 flights. The residual life of individual aircraft in the fleet can be obtained by subtracting the actual consumed life.

## 4. Design of Structural Health Monitoring System

In addition to the real-time monitoring and decision-making of major structural damage, aircraft damage monitoring and life prediction can be carried out off-line on the ground, which can greatly reduce the difficulty of the aircraft and reduce the weight of the aircraft.

### 4.1. Literature Review of Structural Health Monitoring System

For structural health systems or individual aircraft tracking systems, Rodney [34] analyzed different IAT systems, and studied such targets as determining the life expectancy distribution of individual aircraft and evaluating the dispersion coefficient of individual aircraft based on statistical data. In order to improve the accuracy of structural health monitoring, Lee et al. [35] developed a portable strain sensor calibration system, which can achieve static 1% and dynamic 2% accuracy. Main et al. [36] tracked the fatigue of F/A-18 aircraft in service, and studied the importance of lead crack, fracture quantitative analysis, fatigue test and individual aircraft tracking. Liao et al. [37] studied the adaptability of the aircraft digital twin and its potential application in reducing maintenance costs and using potential of the fleet, and improved the current individual aircraft tracking program by quantifying and updating the uncertainty of IAT parameters in the evaluation of aircraft breakdown fatigue life. Renaud et al. [38] developed a quantitative risk assessment tool based on the Bayesian reasoning method. The aircraft ADT method can make better use of IAT data and improve the precision of fatigue life estimation. Through the establishment of a cumulative damage model of the aircraft structure and the implementation of a special inspection program, Li et al. [39] completed the life monitoring of an aircraft in service, reasonably deploying the use of the aircraft and guiding the repair task on it. Smith et al. [40] developed an automation and flexibility program in the A-10 aircraft load/environment spectrum measurement, which promotes an individual aircraft’s tracking procedures and the impact on its structural integrity.

In relation to sensor network design, Bhuiyan et al. [41] studied the position optimization method of a wireless sensor and designed a three-stage sensor installation method, which can reduce the fault probability of a wireless sensor; they also verified the effectiveness and performance of the system simulation by the measured data and physical method. Mustapha et al. [42] carried out the design and optimization of sensor networks, sensor power supply, data analysis and other important parameters, and presented a successful example. Henderson [43] studied the Bayesian calculation model for a SHM sensor in undamaged composite materials, and gave improved estimations for a physical model, sensor node parameters and input source parameters. Ge et al. [44] adopted an intelligent wireless sensor network method and proposed multi-sensor information fusion technology to improve data transmission efficiency and make better use of data. The robustness of polynomial fitting results provided a new idea and method for structural health monitoring. Aiming at the fiber optic sensor, Wang et al. [45] monitored the impact load position and energy of the typical structure of a stiffened wing panel, putting forward the relevant idea of embedding the fiber optic sensor in the composite material in the future. Feng et al. [46] tested the PZT sensor under different damage conditions by using a precision impedance tester, and confirmed the feasibility of monitoring the degree of structural damage by the electro-mechanical impedance (EMI) technique. Xue et al. [47] carried out a leading flight test and other research on the installation of optical fiber sensors in an aircraft, and put forward the prospect of the development of optical fiber sensors being more dense, fast and miniaturized.

In the individual aircraft tracking life prediction model, there has also been various research studies. Terry [48] studied two methods of individual aircraft tracking for crack propagation using gravitational acceleration. For the individual aircraft tracking system of F-4 and A-7D aircraft, the center of gravity overload was adopted. The F-4 used the equivalent S-N curve system to calculate the damage index of the critical position, and the A-7D monitoring method utilized regression analysis to calculate the equivalent reference hours of the critical positions. Both methods correlate the damage of the critical position with that of other critical sites. On the basis of introducing the traditional life assessment method of the U.S. Navy fleet and the individual aircraft tracking method, Nagaraja et al. [49] introduced a life estimation method for P-3C aircraft, carried out a risk assessment based on measured data, and presented an equivalent mathematical formula for failure analysis probability. Finally, crack initiation and crack propagation models were used in aircraft life prediction and fleet management. Dui et al. [50] achieved high precision prediction of crack growth rate and life under random load spectrum by using the model based on the Paris formula and the law of rate analogy. The average spread rate model and the rate analogy rule were used to provide a reliable fatigue life prediction method for individual aircraft life monitoring in serviced aircraft. Based on the variation of vibration response caused by crack growth, the damage index was proposed by Marques et al. [51]. The fatigue life was then estimated using the stress probability density function and the walker crack growth equation: the predicted results were in good agreement with the experimental data. Neerukatti et al. [52] proposed a hybrid prediction model that can accurately predict the crack growth state and residual effective life of aluminum structures. Compared with the simple data-driven or physical model, the prediction accuracy was improved by combining the measured data and physical model. Law et al. [53] studied the comprehensive analysis, numerical and experimental framework of structural integrity, performance testing and life cycle assessment. The Bayesian probability model was used to update the process to verify the potential applicability of damage identification near the critical weld zone of fatigue failure. Chen et al. [54] used UAV and an image processing system to monitor and evaluate the surface degradation of a steel structure, which was an economical and time-critical solution for steel structure health monitoring. By using the finite element model of the wing structure under load, the response parameters of the wing were calculated and the damage characteristics of the wing were revealed by Wan et al. [55]. There were obvious differences between the damaged wing and the normal one. The established wing structure health monitoring system can be used to detect the damage and locate the damage position.

### 4.2. Framework of Structural Health Monitoring System

In general, structural health monitoring systems should include both an on-board system and ground components.

The on-board system collects load-related data from installed sensors, such as strain, acceleration, vibration acceleration, etc., and keeps in communication with the aircraft system: it calls the relevant flight parameters, and stores the load/strain, acceleration and related flight parameters in the load data acquisition equipment in real-time according to the specified format. The data is then used for off-line analysis of load history and life expectancy of critical parts after flight.

The ground system includes damage analysis and data management equipment, relevant data processing and analysis software. As well as having some early-warning risk state function, the system has the ability to give the load history and damage status at each control point for each flight, and also to present the corresponding solution to the service life consumption or structure damage of the aircraft.

**Figure 12:**
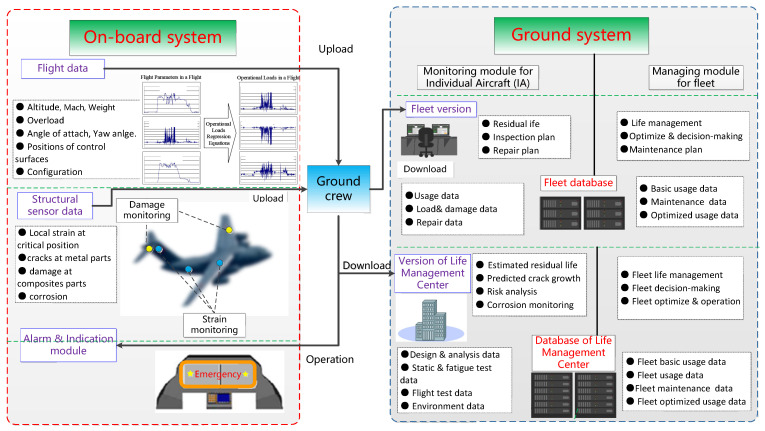
Schematic diagram of structural health monitoring system.

The principle block diagram of the structural health monitoring system is shown in Figure 12.

Different methods should be adopted for the monitoring of new and in-service aircraft or aging aircraft:

- Aircraft under design. At the beginning of the design, a structural health monitoring system is designed, which is implemented with reference to the development process and management methods of airborne finished products.
- In-service aircraft or aging aircraft. The structural health monitoring and life prediction can be realized by the adaptation and modification of critical parts in limited numbers.

## 5. Life Assessment Based on Structural Health Monitoring

All the parameters were recorded continuously by the flight parameter recorder and related sensors during flight, whether in new or in-service aircraft, which makes it possible to determine stress of the critical load at any moment. After determining the stress history of key parts, damage assessment and life prediction can be carried out on this basis.

The main tasks can be summarized as follows.

### 5.1. Selection of Control Points

In the design of the structural health monitoring system, it is necessary to select control points on representative parts and install sensors to reflect the load characteristics of the main parts of the aircraft. Generally speaking, the number of control points should not be too high, yet should be able to reflect the load characteristics of the key parts.

The number of control points in some representative aircraft are: 10 control points for F-35 [15], 40 for A400M [7], 10 for F-22 [14], 10 for F-18 [56], 22 for F-2 [57], 8 for Su-22 [58], and 16 for EF2000 [59]. Taking A400M as an example, the control points mainly cover typical structural parts such as fuselage section and main stiffening frame, wing root and wing beam, wing body connection, vertical tail, horizontal tail, main landing gear connection, and pylon- wing connection.

### 5.2. Selection of Flight Parameters

Life assessment requires the use of the aircraft’s load history. The parameters closely related to load can be selected from hundreds of flight parameters recorded continuously in each flight for structural health monitoring. In general, the following parameters should be included:

- Weight of aircraft.
- Altitude.
- Aircraft speed (ground speed, air speed), Mach number, engine data (thrust, rotational speed, etc.);
- Position of control surfaces (spoiler, aileron, rudder, and elevator).
- Aircraft configuration (opening angle of flaps, slats).
- Angular velocity of pitch, roll, yaw and angular acceleration at the center of gravity.
- Landing gear position.

### 5.3. Load/Stress Conversion Equation

Constructing a high-precision structural load regression model from flight parameters is the key to ensuring the accuracy of load identification and life prediction. A neural network or multiple linear regression method based on limited flight parameters and monitoring sensor data is usually used [60].

The aircraft is subjected to different loads under different mission segments, so it is necessary to establish the transformation equation under different mission segments to reflect the load and stress relationship of the corresponding mission segments. In the case of F-22 aircraft, there are at least three different equations for the same load and stress at the same control point, corresponding to the subsonic, transonic, supersonic and hatch opening states of the aircraft [14].

A simple method is to use linear interpolation based on partial parameters: the accuracy is acceptable to estimate the load of a flight segment during the entire flight, and the coefficient of load between each task segment can be determined by optimal interpolation. Typical equation forms can be written as:

(8) $$Load=C_{1}X_{1}+C_{2}X_{2}+\ldots +C_{n}X_{n}$$

The stress at any specified control point can be obtained by the ‘flight parameters-to-stress’ transformation equation directly from the load and FE result.

The method used to construct the ‘flight parameters-to-stress’ transformation equation is similar to that for the ‘flight parameters-to-load’ transformation equation. Either linear regression or a neural network method is used to construct the transformation equation of load of each task segment through finite flight parameters (Mach number, weight, height...).

### 5.4. Damage and Life Prediction

The damage and life of each flight is expected to be evaluated by an engineering method, which can be the same as the method used in the model design. At present, there are two main methods or calculations: fatigue crack initiation life, and crack propagation life. Which method is adopted depends mainly on the design criteria adopted in the aircraft development. Generally, the fatigue crack initiation analysis is the main method for aircraft designed according to the safety life, and the crack propagation analysis is the main method for aircraft designed according to the damage tolerance.

### 5.5. Result Output

Life assessment based on structural health monitoring is expected to be achieved through integration with ground-based equipment. Flight record data is downloaded to the device by ground crew and processed according to damage assessment software. The output of evaluated results should include: load and stress history, damage and life management parameters and GUI prediction tools.

Damage and life management parameters can be defined by the ratio of flight damage to the average flight damage of the reference spectrum. It is a good choice to set the measured spectrum as reference spectrum, while the design spectrum can also be used when the measured load spectrum is unavailable. The GUI prediction tool is used to track the estimated damage and actual damage of any control point with the change of flights, so it is convenient to track the aircraft life consumption in time.

### 5.6. Verification of Conversion Equation

The principle of life prediction in structural health monitoring, especially the reasonableness of the load conversion equation, must be verified. Generally it can be verified by flight test and full-scale static test:

- Flight tests may be carried out by means of a small batch stage load test aircraft or by means of a subsequent load spectrum test aircraft.
- The load-to-stress equation can be verified by the full-scale static test, but the real load equation cannot be so verified.

## 6. Prospects for the Development of Intelligent Applications in the Future

The development of structural health monitoring is bound to develop towards intelligent application, which may be reflected in the following aspects.

### 6.1. Intelligent Materials/Structures

Intelligent material/structure will be the key research field in structural health monitoring, and the sensing function under intelligent structure will be considered in the short-term. The sensing function and possible actuating device can be integrated into the structure or material, and the sensing device and actuating device are connected through the control device. This combination of sensing–excitation–control can be either a macro-level structure or a micro-level material. Applying this concept to damage monitoring means that the damage and exciter are integrated into the structure–that is, non-destructive testing becomes part of the structure.

### 6.2. Real-Time Life Prediction Model

Most of the current life prediction models are based on physical state, so the life prediction models based on real-time state change of structure cannot be considered. In the future, the dynamic correlation between real-time damage and structural health state data should be considered, a damage prediction model based on structural health should be constructed, and a real-time life prediction and damage prediction model based on a data-driven physical basis should be established by combining theory and numerical analysis.

### 6.3. Realization of Condition-Based Maintenance

Condition-based Maintenance (CBM) is designed to perform maintenance only when there is objective evidence of the need for maintenance, while ensuring the safety and reliability of the equipment and reducing the cost of operation and maintenance.

In order to improve the reliability and reduce the maintenance cost, different maintenance methods are selected scientifically and rationally: a comprehensive set of optimized maintenance methods is formed by the application of structural health monitoring technology, and on the basis of the importance of structure, testing and maintenance level. Such a set might include time-based, post-failure and state maintenance methods. 

### 6.4. Intelligent Damage Recognition

The intelligent recognition of damage is mainly embodied according to the following aspects: no damage, damage location, and damage size. Intelligent identification of structural damage is the basis of aircraft structural life prediction and damage assessment, and is also an important basis for determining whether aircraft structures need maintenance. The intelligent quantitative monitoring of the damage in the structure can be realized through the influence of the signal in each sensor path on the structure damage, combined with the large data statistical analysis model.

### 6.5. Intelligent Diagnosis of Sensor Network

In order to eliminate the misdetection and mis-detection caused by some sensor faults in the sensor network, it will be necessary in future to develop a self-detection method for each sensor network, and to discover and remove the signal of the faulty sensor. At the same time, the information collection in the corresponding area will need to be compensated and collected by other sensors, so as to realize the intelligent diagnosis of the sensor network. The sensor network must be maintained in order to ensure there are never too many damaged sensors in the sensor network, as this would affect the precision of data acquisition required by health monitoring.

### 6.6. Aircraft Digital Twin

In the face of aircraft structure fatigue life management, digital twins will be the future focus and direction of development. In particular, we believe the main research fields in the future will include: the deep fusion of flight parameters; strain sensing and simulation to obtain accurate load data; the development of physical consistency multi-scale analysis theory and software, including the accurate and efficient simulation of damage structures; and the adaptive updating of the reduced order of digital twin models [61]. The development and successful application of the digital twin aircraft structure will be expected to realize early warning, residual life prediction, single-aircraft tracking operation and maintenance of aircraft structure, as well as play an important role in the development of next-generation advanced aircraft.

## 7. Conclusions

1. On the basis of typical cases of its aircraft applications, the basic principle of aircraft structural health monitoring system design is presented, the process of aircraft structural health monitoring and life prediction is put forward, and the elements thereof discussed—namely control point selection, load/strain equation construction, stress calculation of control points, damage calculation, life evaluation and output of results.
2. Research on the IAT of a certain aircraft and the residual life prediction based on the local load spectrum of an aging aircraft are carried out, and the corresponding flow chart is given. The evaluation results of individual aircraft life consumption and the analysis results of residual life of aircraft structures with different reliability can be used for reasonable deployment of aircraft.
3. The development trend of structural health monitoring and life prediction is summarized noting the aspects of intelligent material/structure, real-time life prediction model, realization of condition-based maintenance, intelligent damage identification, self-diagnosis of sensor network and aircraft digital twin.

## Figures and Tables

**Table 1:** 40 representative flights and their occurrences in the reference spectrum.

No.	Name of Representative Flight	Numbers of Representative Flight	No.	Name of Representative Flight	Numbers of Representative Flight
1	A-01	1	21	D-08	14
2	A-02	3	22	D-09	8
3	A-03	1	23	D-12	9
4	A-04	1	24	D-13	6
5	A-05	7	25	E-04	29
6	A-06	1	26	E-05	24
7	A-07	2	27	E-06	52
8	A-08	4	28	F-01	25
9	B-01	1	29	F-06	57
10	B-03	1	30	F-09	12
11	B-04	1	31	F-12	11
12	B-05	1	32	G-01	2
13	B-06	2	33	G-02	1
14	B-07	3	34	G-03	1
15	B-08	5	35	G-04	1
16	B-10	1	36	G-05	2
17	C-01	10	37	G-06	3
18	D-01	15	38	H-01	1
19	D-02	8	39	H-04	1
20	D-03	5	40	H-05	3

**Table 2:** Results of fatigue test under variable spectrum.

Specimens	1# Specimen	2# Specimen	3# Specimen	4# Specimen	5# Specimen	6# Specimen	7# Specimen
Test cycles	503,530	502,281	428,375	400,841	595,248	459,622	423,394

## Data Availability

Not applicable.
